# Investigating the Antioxidant and Immunomodulatory Effects of Quercetin Using Porcine PBMCs as an Inflammatory In Vitro Model

**DOI:** 10.3390/antiox15030358

**Published:** 2026-03-12

**Authors:** Fanni Somogyi, Nikolett Palkovicsné Pézsa, Ákos Jerzsele, Jázmin Németh, Levente Harmat, Orsolya Farkas

**Affiliations:** 1 Department of Pharmacology and Toxicology, University of Veterinary Medicine, 1078 Budapest, Hungary; somogyi.fanni@univet.hu (F.S.); jerzsele.akos@univet.hu (Á.J.); nemeth.jazmin@student.univet.hu (J.N.); farkas.orsolya@univet.hu (O.F.); 2 National Laboratory of Infectious Animal Diseases, Antimicrobial Resistance, Veterinary Public Health and Food Chain Safety, University of Veterinary Medicine, 1078 Budapest, Hungary; 3 Experimental Farm, University of Veterinary Medicine Budapest, 2225 Üllő, Hungary; harmat.levente@univet.hu

**Keywords:** porcine PBMC, quercetin, *Salmonella* Typhimurium LPS, *Salmonella* Enteritidis LPS, *Escherichia coli* LPS, ConA, PHA, oxidative stress, proinflammatory cytokines

## Abstract

As the human population continues to grow, the demand for pork increases, and the management of infectious diseases in swine from a One Health standpoint is becoming more important than ever. To prevent antimicrobial use as much as possible, the search continues for alternative substances that can aid in mitigating oxidative stress and inflammation, which are cornerstones of infectious disease. In this study, we stimulated porcine peripheral mononuclear blood cells (pPBMCs) with either bacterial lipopolysaccharides (LPS) of different origin (*Salmonella* Typhimurium, *Salmonella* Enteritidis and *E. coli*), or the plant lectins concanavalin A (ConA) and phytohemagglutinin (PHA) to create an in vitro inflammatory model. Quercetin, a flavonoid with well documented positive effects, was used with the aim of decreasing oxidative stress and the production of the inflammatory cytokines IL-6 and IL-8. Oxidative stress was successfully induced in the pPBMCs by all stimulants (except for *S.* Enteritidis LPS), along with IL-6 production (except for *E. coli* LPS); IL-8 production was only induced by treatment with LPS. While quercetin had an antioxidant effect on the pPBMCs, it did not reduce IL-6 or IL-8 levels under the conditions tested and even had a pro-inflammatory effect by increasing IL-8 production when combined with LPS. To gain a deeper understanding of the immunomodulatory effects of quercetin on pPBMCs, further studies should be conducted to measure the production of additional pro- and anti-inflammatory cytokines, including TNF-α, IL-10, and IL-1β.

## 1. Introduction

The swine industry is one of the most important food-producing industries, and it continues to grow as the global human population and the demand for pork increases. Large-scale pig farms often house thousands of animals indoors in high densities, which enables the accumulation and rapid transmission of pathogens, often resulting in infectious diseases [1,2,3]. Certain age groups during the rearing process are especially predisposed to infections; for example, weaned piglets often develop post-weaning diarrhoea (PWD) due to several environmental, nutritional and psychological stressors, and this can result in a significant loss of growth performance [4]. Prevention of these pathologies is important for several reasons, the main ones being animal welfare, economic productivity, food safety and public health in the case of zoonotic pathogens, and antimicrobial resistance [2,5]. In accordance with the One Health concept, the close relationship between humans and farm animals greatly increases the prevalence of zoonotic infections in humans. Nearly all stages of animal food production (rearing and slaughtering animals; retailing, cooking and consuming animal products) can expose humans to zoonotic pathogens or aid the transmission of antibiotic resistance genes between livestock and humans. Therefore, from a One Health perspective, it is key to prevent infectious diseases in farm animals, and reduce antimicrobial use [6,7].

Oxidative stress and inflammation are integral parts of the immune system’s response to infectious diseases. Oxidative stress, or the disturbance of redox homeostasis, is a complex phenomenon characterised by the overproduction of reactive oxygen species (ROS), which can lead to cellular damage, necessitating the need for protective antioxidant systems [8,9]. ROS play a multifaceted role in the body, as they are products of normal cellular metabolism at low or moderate concentrations, taking part in several intracellular signalling systems, as well as cell growth, differentiation, etc. They are also involved in nearly all stages of the inflammatory response. During the initial stages of inflammation, ROS help with the recruitment of leukocytes to the site of tissue injury by increasing vascular permeability and leukocyte extravasation, while also acting as microbicidal agents, produced by phagocytes in the form of respiratory bursts [10,11,12]. If, however, ROS are produced in such an excess that cannot be compensated by the antioxidant systems, oxidative stress occurs, during which ROS can damage important components of the cell such as lipids, proteins and DNA, leading to impaired cell functionality and, potentially, to cell death [10,13]. ROS (as well as other agents like LPS and TNF-α) are modulators of the NF-κB signalling pathway, capable of activating it and thus contributing to the production of proinflammatory cytokines such as IL-1β, IL-6 and IL-8 [14,15,16]. The immune response triggered by infectious agents needs to be controlled tightly: a response too light can enable the pathogen to escape elimination and compromise the survival of the organism; on the other hand, uncontrolled and persistent oxidative stress and inflammation can lead to acute organ damage or chronic inflammation [17,18].

The practice of feeding antibiotics to pigs in sub-therapeutic levels to promote growth and prevent infectious diseases, which dates to the 1950s, has contributed to the rise of antimicrobial resistance in pathogenic bacteria, a major public health hazard. Nowadays, the use of antibiotics is heavily controlled: in many countries (e.g., EU member states, USA), using them for growth promotion in livestock has been banned for several years now, and their pro- and metaphylactic use is also restricted. Therefore, there is a growing need for alternative substances that contribute to the health and performance of farm animals, such as organic acids, enzymes, probiotics, prebiotics, symbiotics, antimicrobial peptides and phytochemicals [19,20,21]. The latter group includes flavonoids, which are plant secondary metabolites with a polyphenolic structure. They can be found widely throughout the plant kingdom and have long been studied for their positive biological properties, such as antioxidant and anti-inflammatory effects [22,23,24].

Quercetin (Q) is a polyphenol that belongs to the flavonol subclass of flavonoids, and is found in a wide variety of fruits, vegetables and plant-derived beverages [22]. It has attracted considerable attention due to its well-documented positive effects, including antioxidant and anti-inflammatory properties. As an antioxidant, it acts through several complementary mechanisms: it directly scavenges ROS and binds transition metal ions, thereby inhibiting lipid peroxidation. In addition, it enhances endogenous antioxidant defence systems by increasing the activity of antioxidant enzymes such as catalase (CAT), superoxide dismutase (SOD), and glutathione peroxidase (GPx). Quercetin also reduces ischemia-related oxidative reperfusion injury by interfering with inducible nitric oxide synthase (iNOS) and the xanthine oxidase pathway. Furthermore, it stabilizes cellular membranes by acting as a calmodulin antagonist, and modulates redox-sensitive signalling cascades, including the p38 MAPK, Nrf2, and AMPK pathways [25,26,27,28].

In terms of anti-inflammatory activity, quercetin has been shown to inhibit the lipopolysaccharide (LPS)-induced production of pro-inflammatory cytokines such as TNF-α, IL-8 and IL-1α, as well as Th2-derived IL-4 and IL-1 stimulated IL-6. Quercetin attenuates TNF-α-mediated inflammation by preventing the activation of nuclear factor-κB (NF-κB) and by enhancing the activity of peroxisome proliferator-activated receptor-γ (PPARγ), which antagonizes NF-κB signalling. In addition, it reduces the expression of the inflammation-related enzymes cyclooxygenase (COX), lipoxygenase (LOX) and matrix metalloproteinases (MMPs). Quercetin also stabilizes mast cells and basophils, thereby decreasing histamine release [29,30,31]. Most of these findings have been reported in studies using human or rodent cell models, while research on porcine immune cells is still limited. This knowledge gap highlights the importance of investigating the effects of quercetin using porcine cell models, such as peripheral blood mononuclear cells (PBMCs) of porcine origin (pPBMCs).

PBMCs represent a diverse population of peripheral blood cells isolated from whole blood. The primary components are lymphocytes (including T helper (Th) CD4+, T cytotoxic (Tc) CD8+, T regulatory (Treg), gamma-delta (γδ) T cells, B and natural killer (NK) cells), as well as monocytes and plasmacytoid dendritic cells (pDCs). Being key elements of both innate and adaptive immunity, these cells can be used in a wide array of scientific research, including modelling immune responses to viral and bacterial infections, studying chronic diseases and inflammation, as well as testing new therapeutic strategies such as antioxidants and immunomodulators [32,33]. Using pPBMCs in experiments can yield valuable results for both veterinary and human medicine, as pigs are a significant species in veterinary translational research, sharing many anatomical, genetic and immunological similarities with humans [32].

In our study, we examined the antioxidant and anti-inflammatory activity of quercetin in pPBMCs challenged with LPS of different bacterial origins (*Salmonella enterica* ser. Typhimurium, *Salmonella enterica* ser. Enteritidis and *Escherichia coli*), as well as the plant lectins concanavalin A (ConA) and phytohemagglutinin (PHA). LPS, found in the outer cell membrane of Gram negative bacteria, is a pathogen-associated molecular pattern (PAMP) that activates monocytes and B cells through pattern recognition receptors (PRRs), producing a rapid innate immune response [34,35]. ConA (a glycoprotein extracted from Jack bean, *Cannavalia ensiformis*), and PHA (the lectin extract from the red kidney bean, *Phaseolus vulgaris*) are non-specific T cell mitogens; they stimulate the metabolic activity and cell division of T cells and other mononuclear cells, and the production of a wide range of pro- anti anti-inflammatory cytokines [34,36,37]. After activating the cells with LPS, ConA or PHA, and treating them with quercetin, we observed the intracellular ROS production of the cells, as well as their secretion of two pro-inflammatory cytokines, interleukin-6 (IL-6) and interleukin-8 (IL-8).

## 2. Materials and Methods

### 2.1. Chemicals

Dimethyl-sulfoxide (DMSO, Hybri-Max, sterile filtered, BioReagent, suitable for hybridoma (≥99.7%), Histopaque (sterile-filtered, density: 1.077 g/mL), quercetin (Q; ≥95%, HPLC grade) and lipopolysaccharides (LPS) (derived from *Salmonella enterica* ser. Typhimurium L6143, *Salmonella enterica* ser. Enteritidis L770 and *Escherichia coli* O111:B4, suitable for cell culture) were purchased from Sigma-Aldrich–Merck (Darmstadt, Germany). Concanavalin A (ConA), phytohemagglutinin (PHA; M form) and Mammalian Protein Extraction Reagent (M-PER) were purchased from ThermoFischer Scientific (Rockford, IL, USA). RPMI 1640 W/L-glutamine was purchased from Biosera–BioTech Hungary (Szigetszentmiklós, Hungary). Red Blood Cell Lysis Buffer was purchased from Roche Diagnostics (Mannheim, Germany).

### 2.2. Porcine Blood Sampling and Isolation of pPBMCs

For the isolation of white blood cells, porcine blood was used. The blood samples were residual material obtained from routine veterinary diagnostic procedures and were not collected specifically for the purposes of this study; therefore, in accordance with the applicable regulations, their use does not constitute an animal experiment. A formal certificate of exemption was issued by the Head of the Animal Welfare Committee of the University of Veterinary Medicine, Budapest, confirming that the use of residual diagnostic material in this study does not require ethical approval. Blood samples from three pigs were collected by jugular venipuncture into vacuum tubes (10 mL) containing K_2_ EDTA. Porcine PBMCs were isolated from whole blood within 2 h of collection by density gradient centrifugation with Histopaque (1.077 g/mL). Whole blood was layered onto Histopaque solution (3 mL each) and centrifuged in 15 mL tubes (400× *g*, 23 °C, 30 min). Cells were harvested from the interface layer within 10 min after centrifugation (approx. 1 mL/tube) and washed once using Red Blood Cell Lysis Buffer (1 mL) and PBS (10 mL). After centrifugation (100× *g*, 23 °C, 10 min), the supernatant was discarded, and cells were resuspended in supplemented RPMI medium (50 mL FBS and 5 mL penicillin-streptomycin were added to 500 mLs of RPMI 1640 W/L-glutamine). Cells were counted using a hemocytometer, seeded at a concentration of 10^5^ cells/mL onto 24-well plates or 96-well plates depending on the type of experiment, and incubated for 24 h before treatment (37.5 °C, 5% CO_2_).

PBMCs were isolated from three pigs overall and processed independently without pooling. Depending on sample availability and assay requirements, not all experiments were performed with cells from all animals. Statistical comparisons were conducted within each experiment using technical replicates for each treatment condition.

### 2.3. Measurement of the Metabolic Activity of pPBMCs

The influence of the three types of LPS used, as well as ConA, PHA and Q on the viability of pPBMCs at different concentrations was tested using the Cell Counting Kit-8 assay (CCK-8, Sigma-Aldrich, Darmstadt, Germany). This assay utilizes the ability of the tetrazolium salt WST-8 to be reduced to a water-soluble formazan dye by intracellular dehydrogenases. The amount of formazan dye generated is directly proportional to the number of living cells and their metabolic activity. Porcine PBMCs were seeded onto 96-well plates at a concentration of 10^5^ cells/mL; 100 μL of supplemented RPMI medium containing pPBMCs was added to each well. After incubation for 24 h (37.5 °C, 5% CO_2_), 100 μL of treatment medium was added to each well. The treatment groups are described in Table 1. Quercetin was dissolved using sterile DMSO; final DMSO concentration in wells was 0.25 *V*/*V*% in the case of treatment with Q-25, and 0.125 *V*/*V*% in the case of treatment with Q-50. Following another 24 (in the case of Q and LPS) or 48 h (in the case of ConA and PHA) of incubation (37.5 °C, 5% CO_2_), cells were treated with CCK-8 reagent (10 μL in each well), and incubated for 2 h (37.5 °C, 5% CO_2_). Absorbance was measured at a wavelength of 450 nm using a Spectramax iD3 instrument (Molecular Devices, San Jose, CA, USA).

### 2.4. Determination of Intracellular ROS Production of pPBMCs

To evaluate the effect of LPS, ConA, PHA, Q and their combinations on the intracellular reactive oxygen species (IC ROS) production of pPBMCs, the DCFH-DA method was used. In the presence of intracellular ROS, the 2′, 7′-dichloro-dihydrofluorescein diacetate (DCFH-DA) dye (Sigma-Aldrich, Budapest, Hungary) is oxidized to the highly fluorescent form dichloro-fluorescein (DCF). Cells were seeded onto 24-well plates at a concentration of 10^5^ cells/mL; 500 μL of supplemented RPMI medium containing pPBMCs was added to each well. After incubation for 24 h (37.5 °C, 5% CO_2_), 500 μL of treatment medium was added to each well. In addition to the treatment groups described in Table 1, combination treatment groups summarized in Table 2 were also used. The cells were incubated for 24 h (in the case of treatment with LPS and LPS + Q combinations) or 48 h (in the case of treatment with ConA, PHA and ConA/PHA + Q combinations) (37.5 °C, 5% CO_2_) and then treated with the DCFH-DA reagent (4 μM; 500 μL in each well). After 1 h of incubation, the reagent was removed, and the cells were lysed and scraped using M-PER and PBS (200 μL/well). The supernatant containing the lysed cells was pipetted into Eppendorf tubes and centrifuged (700× *g*, 4 °C, 10 min). After that, 100 μL of supernatant from each sample was added to a 96-well plate. A Spectramax iD3 instrument (Molecular Devices, San Jose, CA, USA) was used to measure the fluorescence at an excitation wavelength of 480 nm and an emission wavelength of 530 nm.

### 2.5. IL-6 and IL-8 Determination with ELISA

For the enzyme-linked immunosorbent assay (ELISA), cells were seeded onto 24-well plates after isolation, and treatments were performed as described in Section 2.4. Before cells were treated with the DCFH-DA reagent, 500 μL of supernatant was collected from each well, pipetted into Eppendorf tubes and frozen at −80 °C until later use. After thawing the samples at room temperature, the IL-6 and IL-8 concentration was determined by porcine-specific ELISA Kits (Sigma-Aldrich, Darmstadt, Germany) according to the manufacturer’s instructions.

### 2.6. Statistics

Statistical analysis of the data obtained during the cell culture experiments was performed using R software (version 4.4.3; R Foundation for Statistical Computing, Vienna, Austria). To enable data comparability, a control percentage (%) was used: the mean concentration of the control cells served as the reference point at 100%, against which values from various treatment groups were compared. Prior to hypothesis testing, mean values and standard deviations were computed across all treatment groups, and data normality was assessed using the Shapiro–Wilk test. Statistical comparisons were performed separately for each stimulus condition to evaluate the effect of different quercetin concentrations. For this purpose, one-way analysis of variance (ANOVA) followed by Tukey’s post hoc multiple comparison test was applied. Technical replicates were averaged for each animal prior to statistical analysis, and each pig was considered one biological replicate (*n* = 3). A *p*-value < 0.05 was considered statistically significant. For clarity, results presented in the figures and text include specific significance levels (e.g., *p* < 0.05, *p* < 0.01, *p* < 0.001).

## 3. Results

### 3.1. Porcine PBMC Metabolic Activity

**Figure 1:**
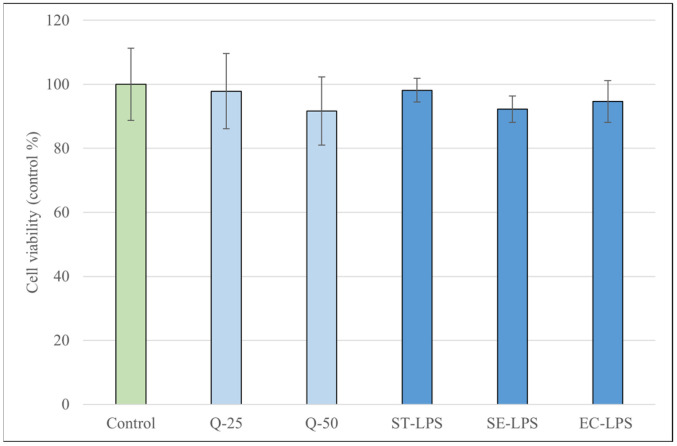
Metabolic activity of porcine peripheral blood mononuclear cells (pBMCSs) after treatment for 24 h with different concentrations of quercetin (Q, 25 μM and 50 μM) and lipopolysaccharide derived from *Salmonella enterica* ser. Typhimurium L6143, 1 μg/mL (ST-LPS), *Salmonella enterica* ser. Enteritidis L770, 1 μg/mL (SE-LPS) and *Escherichia coli* O111:B4, 1 μg/mL (EC-LPS). Data are shown as means with standard deviations in control percentage and are statistically compared to those of the control group (*n* = 6/group).

**Figure 2:**
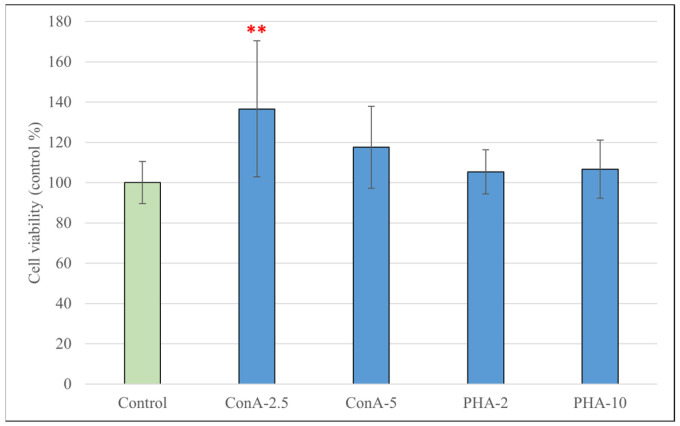
Metabolic activity of porcine peripheral blood mononuclear cells (pBMCSs) after treatment for 48 h with different concentrations of concanavalin A (ConA, 2.5 μg/mL and 5 μg/mL) and phytohemagglutinin A (PHA, 2 *V*/*V*% and 10 *V*/*V*%). Data are shown as means with standard deviations in control percentage and are statistically compared to those of the control group (*n* = 6/group; ** *p* < 0.01).

To determine the proper concentration of the three different types of LPS, along with ConA, PHA and Q for the experiments without the reduction in metabolic activity, we used the Cell Counting Kit-8 assay. None of the treatments resulted in a significant reduction in metabolic activity after 24 (in the case of quercetin and LPS) or 48 h (in the case of ConA and PHA) of incubation; moreover, ConA at a lower concentration (2.5 μg/mL) increased the metabolic activity of the cells compared to the control group (*p* < 0.01) (Figure 1 and Figure 2).

### 3.2. Production of IC ROS in pPBMCs After Treatments with Different Types of LPS and Different Concentrations of ConA, PHA and Q

**Figure 3:**
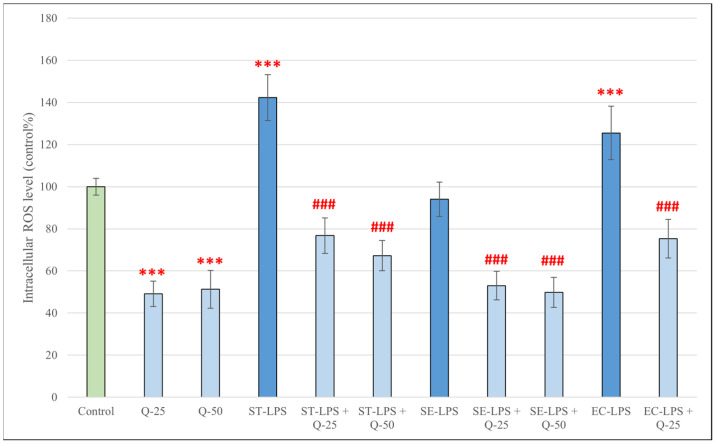
Intracellular reactive oxygen species levels in pPBMCs after treatment for 24 h with quercetin (Q, 25 μM and 50 μM), lipopolysaccharide derived from *Salmonella enterica* ser. Typhimurium L6143, 1 μg/mL (ST-LPS), *Salmonella enterica* ser. Enteritidis L770, 1 μg/mL (SE-LPS) and *Escherichia coli* O111:B4, 1 μg/mL (EC-LPS), and their combinations. Data are shown as means with standard deviations in control percentage (*n* = 8/group). Data from treatment with only Q-25, Q-50, ST-LPS, SE-LPS or EC-LPS are statistically compared to those of the control group (*** *p* < 0.001); data from combination treatments are statistically compared to treatment with only ST-LPS, SE-LPS or EC-LPS (### *p* < 0.001).

Compared to the control group, the IC ROS levels of pPBMCs showed a significant increase after treatment with LPS derived from *S.* Typhimurium and *E. coli* for 24 h (*p* < 0.001) (Figure 3). LPS derived from *S.* Enteritidis, however, did not have the same effect. Treatment with quercetin alone resulted in a significant decrease in IC ROS levels (*p* < 0.001). All treatment combinations of LPS and quercetin significantly decreased the production of IC ROS compared to treatment with LPS alone (*p* < 0.001).

**Figure 4:**
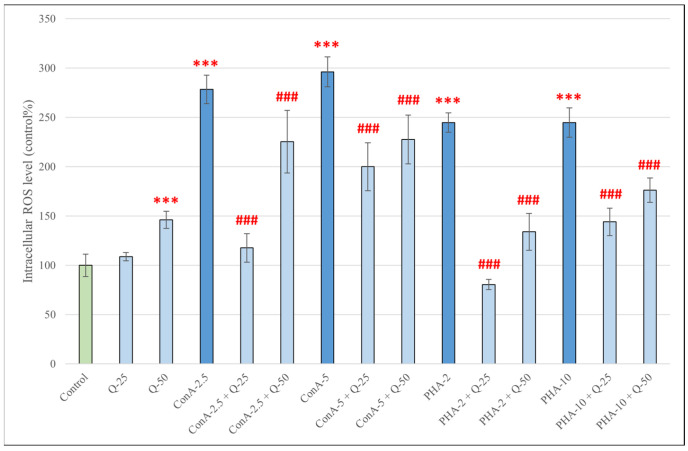
Intracellular reactive oxygen species levels in pPBMCs after treatment for 48 h with quercetin (Q, 25 μM and 50 μM), concanavalin A (2.5 and 5 μg/mL), phytohemagglutinin A (2 and 10 *V*/*V*%) and their combinations. Data are shown as means with standard deviations in control percentage (*n* = 8/group). Data from treatment with only Q-25, Q-50, ConA-2.5, ConA-5, PHA-2 or PHA-10 are statistically compared to those of the control group (*** *p* < 0.001); data from combination treatments are statistically compared to treatment with only ConA-2.5, ConA-5, PHA-2 or PHA-10 (### *p* < 0.001).

Treatment with both concentrations of ConA (2.5 and 5 μg/mL) and PHA (2 and 10 *V*/*V*%) for 48 h significantly increased the IC ROS production in pPBMCs (*p* < 0.001) (Figure 4). Interestingly, when applied to the cells alone, quercetin at 50 μM significantly increased IC ROS production compared to the control group (*p* < 0.001), but after simultaneous treatment with ConA or PHA, both concentrations of quercetin managed to significantly decrease IC ROS levels compared to treatment with ConA or PHA alone (*p* < 0.001).

### 3.3. Production of IL-6 in pPBMCs After Treatments with Different Types of LPS and Different Concentrations of ConA, PHA and Q

**Figure 5:**
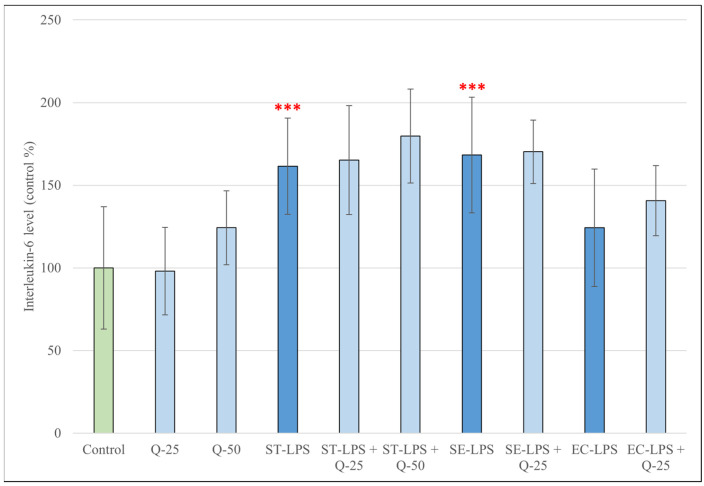
Concentrations of interleukin-6 (IL-6) produced by pPBMCs after treatment for 24 h with quercetin (Q, 25 μM and 50 μM), lipopolysaccharide derived from *Salmonella enterica* ser. Typhimurium L6143, 1 μg/mL (ST-LPS), *Salmonella enterica* ser. Enteritidis L770, 1 μg/mL (SE-LPS) and *Escherichia coli* O111:B4, 1 μg/mL (EC-LPS), and their combinations. Data are shown as means with standard deviations in control percentage (*n* = 8/group). Data from treatment with only Q-25, Q-50, ST-LPS, SE-LPS or EC-LPS are statistically compared to those of the control group (*** *p* < 0.001); data from combination treatments are statistically compared to treatment with only ST-LPS, SE-LPS or EC-LPS.

The IL-6 production of pPBMCs after treatment for 24 h with different types of LPS, different concentrations of Q and their combinations are shown in Figure 5. LPS derived from *S.* Typhimurium and *S.* Enteritidis caused a significant increase in IL-6 levels compared to the control group (*p* < 0.001). LPS derived from *E. coli*, and quercetin at 25 or 50 μM, however, did not cause significant changes. Simultaneous treatment with LPS and quercetin did not result in significant changes compared to treatment with LPS alone.

**Figure 6:**
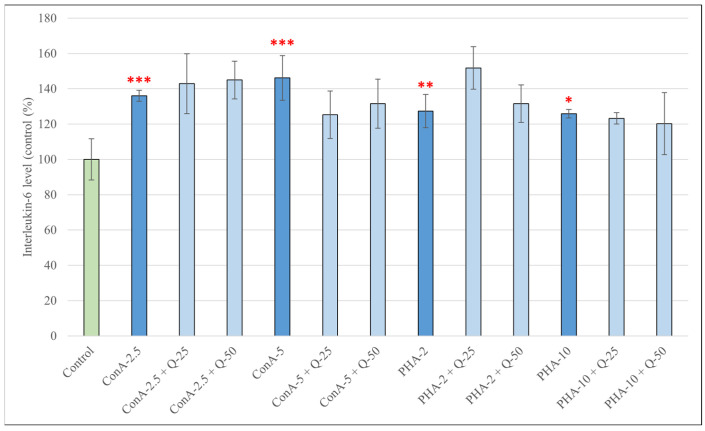
Concentrations of interleukin-6 (IL-6) produced by pPBMCs after treatment for 48 h with concanavalin A (2.5 and 5 μg/mL), phytohemagglutinin A (2 and 10 *V*/*V*%) and their combinations with quercetin (Q, 25 μM and 50 μM). Data are shown as means with standard deviations (*n* = 3/group). Data from treatment with only Q-25, Q-50, ConA-2.5, ConA-5, PHA-2 or PHA-10 are statistically compared to those of the control group (* *p* < 0.05; ** *p* < 0.01; *** *p* < 0.001); data from combination treatments are statistically compared to treatment with only ConA-2.5, ConA-5, PHA-2 or PHA-10.

The IL-6 production of pPBMCs after treatment for 48 h with different concentrations of ConA, PHA and their combinations with Q are shown in Figure 6. Stimulation with both concentrations of ConA (2.5 and 5 μg/mL) and PHA (2 and 10 *V*/*V*%) caused a significant increase in the production of IL-6 compared to the control group (*p* < 0.001 in both cases of ConA; *p* < 0.01 in the case of PHA, 2 *V*/*V*%; *p* < 0.05 in the case of PHA, 10 *V*/*V*%). Simultaneous treatment with ConA or PHA and quercetin, however, did not result in significant changes compared to treatment with ConA or PHA alone.

### 3.4. Production of IL-8 in pPBMCs After Treatments with Different Types of LPS and Different Concentrations of ConA, PHA and Q

**Figure 7:**
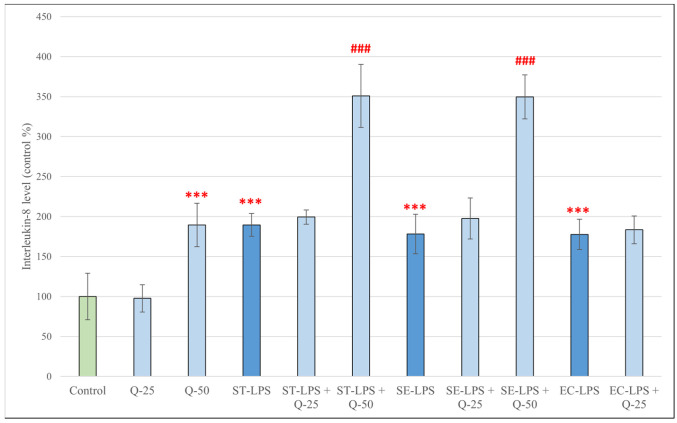
Concentrations of interleukin-8 (IL-8) produced by pPBMCs after treatment for 24 h with quercetin (Q, 25 μM and 50 μM), lipopolysaccharide derived from *Salmonella enterica* ser. Typhimurium L6143, 1 μg/mL (ST-LPS), *Salmonella enterica* ser. Enteritidis L770, 1 μg/mL (SE-LPS) and *Escherichia coli* O111:B4, 1 μg/mL (EC-LPS), and their combinations. Data are shown as means with standard deviations (*n* = 8/group). Data from treatment with only Q-25, Q-50, ST-LPS, SE-LPS or EC-LPS are statistically compared to those of the control group (*** *p* < 0.001); data from combination treatments are statistically compared to treatment with only ST-LPS, SE-LPS or EC-LPS (### *p* < 0.001).

Treatment of pPBMCs with all three types of LPS, as well as quercetin at 50 μM resulted in a significant increase in IL-8 levels compared to the control group (*p* < 0.001) (Figure 7). Simultaneous treatment with LPS derived from *S.* Typhimurium or *S.* Enteritidis and quercetin at 50 μM also resulted in significantly increased IL-8 production (*p* < 0.001); this was not observed in the case of simultaneous treatment with LPS and quercetin at 25 μM.

**Figure 8:**
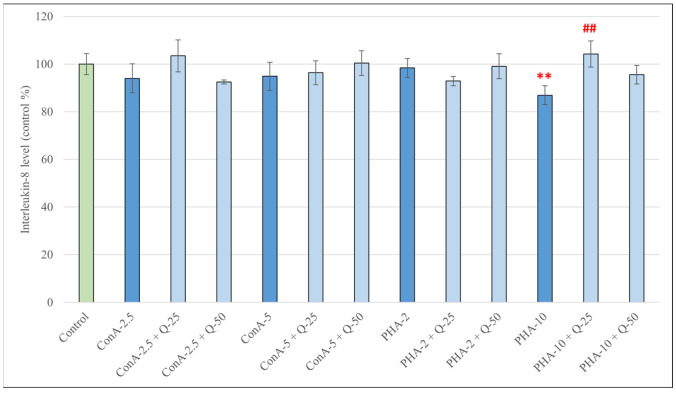
Concentrations of interleukin-8 (IL-8) produced by pPBMCs after treatment for 48 h with concanavalin A (2.5 and 5 μg/mL), phytohemagglutinin A (2 and 10 *V*/*V*%) and their combinations with quercetin (Q, 25 μM and 50 μM). Data are shown as means with standard deviations (*n* = 3/group). Data from treatment with only Q-25, Q-50, ConA-2.5, ConA-5, PHA-2 or PHA-10 are statistically compared to those of the control group (** *p* < 0.01) data from combination treatments are statistically compared to treatment with only ConA-2.5, ConA-5, PHA-2 or PHA-10 (## *p* < 0.01).

Treatment of pPBMCs with different concentrations of ConA or PHA did not result in a significant increase in IL-8 production compared to the control group; treatment with PHA at 10 *V*/*V*% resulted in a decrease in IL-8 levels (*p* < 0.01) (Figure 8). Out of the combination treatments, only cells treated with PHA at 10 *V*/*V*% and quercetin at 25 μM produced significantly more IL-8 compared to cells treated with PHA alone (*p* < 0.01).

## 4. Discussion

As the demand for pork increases together with the growing human population, preventing and managing porcine infectious diseases is key from a One Health standpoint. Avoiding such diseases helps to maintain animal welfare, decreases treatment costs and economic loss, and improves food hygiene, preventing zoonoses in humans. Finding alternative substances that can aid in the management of porcine infectious diseases and can mitigate the use of antibiotics is crucial to achieve this [38]. Using in vitro models such as porcine PBMCs to test these substances is in accordance with the 3R principle of animal experimentation (replacement, reduction, refinement) [39]. These models are also useful in translational research, as pigs are a favorable species for modelling infectious disease and immunological processes in humans; the porcine and human genome show many similarities among immune-related proteins, and the immune response of the two species are highly comparable [40]. Our aim was to use a pPBMC model to study the antioxidant and immunomodulatory effects of quercetin after stimulating the cells with either LPS, ConA or PHA.

The different incubation times (24 h for LPS; 48 h for ConA/PHA) were justified by the different kinetics of the stimulatory agents used in the study. LPS primarily triggers rapid activation of the innate immune response (macrophage/monocyte pathways), and changes measured in cells and secreted mediators (e.g., ROS, IL-6/IL-8) develop within a short period of time (hours to 24 h); therefore, the 24 h endpoint after LPS treatment is appropriate and common in the literature [41,42]. In contrast, ConA and PHA are polyclonal T-cell mitogens that trigger antigen-independent T-cell activation and proliferation: T-cell division and cytokine responses associated with proliferation and activity often peak within 48–72 h, so in the case of ConA/PHA, a 48 h measurement may be better for detecting a more stable, marked signal [43,44]. Nevertheless, a comprehensive kinetic analysis including multiple time points for all stimulants would provide additional insight into the temporal dynamics of these responses and should be considered in future studies.

When assessing the metabolic activity of the cells using the CCK-8 assay, our results showed that none of the used concentrations or types of Q, LPS and PHA resulted in a significant change in metabolic activity. In the case of ConA, a concentration of 2.5 µg/mL significantly increased the CCK-8 signal, a result not observed at a concentration of 5 µg/mL. Goyarts et al. showed that when stimulating porcine blood lymphocytes with ConA, the highest effect occurred using concentrations within a range of 1.25 to 5 µg/mL, and using higher concentrations resulted in a decrease in stimulation [45]. Other studies using pPBMCs have proven that ConA at a concentration of 5 µg/mL works reliably as a T-cell mitogen [46,47]. Our findings indicate that while there is an ideal concentration window for activation with ConA, most likely between 2.5 and 5 µg/mL, responses may vary, as they could also be influenced by differences in the donor animal, culture conditions, etc. These results confirm that the substances used for treating the cells did not have a marked damaging effect which could have contributed to secondary changes in ROS or IL-6/IL-8 levels.

When measuring the levels of intracellular ROS by using the DCFH-DA method, we observed that treatment with all used concentrations of ConA and PHA resulted in a significant increase in ROS levels; the same was true for treatment with LPS derived from *Salmonella* Typhimurium and *E. coli*. LPS derived from *Salmonella* Enteritidis, however, did not result in an increase in ROS production. A possible explanation for the differences in response to different types of LPS could be that *S.* Typhimurium and *E. coli* are highly prevalent, immunostimulatory and pathogenic bacteria in swine [48,49], whereas, while pigs can be carriers of S. Enteritidis, it is less frequently isolated in this species, is only rarely associated with disease, and might not trigger the same magnitude of immune response [50,51]. Although all LPS preparations exhibited comparable endotoxin activity (≥500,000 EU/mg), structural differences between serovars may contribute to differential ROS induction. Treatment with both concentrations of quercetin (25 and 50 μM) alone for 24 h resulted in a significant decrease in intracellular ROS levels; however, after treatment for 48 h, although it did not cause significant changes at 25 μM, quercetin significantly elevated ROS levels at 50 μM. This time- and dose-dependent pro-oxidant capability of quercetin has been described previously. Giordano and Lionetto reported predominantly antioxidant effects of quercetin (5–50 μM) in HeLa cells under both basal and pro-oxidant conditions after 1 h of incubation [52]; in contrast, Kim et al. found that quercetin at 50 μM increased ROS generation in HCT116 colon cancer cells in a time-dependent manner during 12 h of incubation [53]. An explanation for this could be that at higher concentrations or prolonged incubation, quercetin can undergo auto-oxidation, generating metabolites that may promote ROS formation. Boots et al. describes this as “the quercetin paradox”: during the neutralization of H_2_O_2_, quercetin is oxidized into quercetin-quinones, which will arylate GSH and protein thiol groups, leading to GSH consumption, an increase in cytosolic free calcium concentration and LDH leakage [54]. Immune cells are particularly sensitive to redox modulation; thus, prolonged high-dose exposure may exceed the cellular antioxidant buffering capacity of pPBMCs, resulting in measurable ROS accumulation. However, when combined with LPS, ConA or PHA, both concentrations of quercetin significantly decreased intracellular ROS, alleviating the oxidative stress caused by the stimulatory compounds. This powerful antioxidant ability of quercetin against ROS-inducing stimuli has been reported in many other experiments, such as ones using human [55,56] and canine [57] PBMCs, as well as porcine intestinal cells [58,59].

When measuring the IL-6 production of the cells, we have found that, except for LPS derived from *E. coli*, all stimulatory compounds have managed to significantly increase IL-6 levels. These findings mostly correspond with other experiments using porcine immune cells, which have shown a reliable increase in IL-6 production after treatment with LPS [60,61]. The fact that *E. coli* LPS did not induce a strong IL-6 response was unexpected. Scamurra et al. showed that while porcine PBMCs are a significant source of IL-6 after stimulated with *E. coli* LPS, porcine alveolar macrophages are much less consistent when it comes to IL-6 production, as there is considerable variance between donors: cells from 14 of the 20 donor animals were “low responders”, with LPS having little or no effect on IL-6 mRNA levels, while successfully inducing IL-1β, IL-8 and TNF-α [35]. It is possible that during our experiments, donor variance had contributed to the absence of *E. coli* LPS-induced significant IL-6 production. Treatment with quercetin, either alone or in combination with LPS, ConA or PHA did not result in significant changes in IL-6 levels. This was also a puzzling result, as the in vitro IL-6-decreasing effect of quercetin has been shown using many cell types, such as human PBMCs [62], human neutrophils [63] and porcine IPEC-J2 enterocytes [64]. A possible explanation is that although quercetin has been shown to modulate NF-κB and other inflammatory pathways [65], IL-6 expression is regulated by a complex network of transcription factors acting on multiple cis-regulatory elements of the IL-6 promoter (such as NF-κB, AP-1, C/EPB, Sp1, etc.) [66]; this can especially be the case in a mixed population of immune cells such as PBMCs. A limitation of the present study is the limited number of donor animals used for PBMC isolation. Although experiments were performed with independent samples and multiple technical replicates, additional studies including a larger number of animals would be valuable to further evaluate potential inter-individual variability in IL-6 responses.

In our experiments, IL-8 production showed a markedly different pattern compared with IL-6. All three types of LPS significantly increased IL-8 levels, consistent with the well-established role of LPS as a potent inducer of IL-8 transcription in mammalian immune cells [67]. In contrast, ConA and PHA at 2 *V*/*V*% had no significant effect on IL-8, whereas high-dose PHA (10 *V*/*V*%) unexpectedly reduced IL-8 production. This likely reflects the cell-type specificity of IL-8 production: IL-8 is primarily produced by monocytes/macrophages, dendritic cells and epithelial cells in response to innate stimuli like bacteria or LPS [68,69], whereas T-cell mitogens such as ConA and lower PHA concentrations predominantly activate lymphocytic pathways that do not directly drive IL-8 expression. Quercetin alone at 25 μM did not affect IL-8 levels, whereas at 50 μM, it significantly increased IL-8 both alone and in combination with LPS. This finding contrasts with previous reports demonstrating IL-8 suppression in porcine PBMCs [70], human PBMCs [56] and porcine IPEC-J2 cells [64]. However, important methodological differences should be considered in addition to species and cell-type variation. In the cited studies, quercetin was applied at lower concentrations and/or for shorter incubation periods (e.g., 50 μM for 3 h in pPBMCs; 10 μM for 24 h in hPBMCs; 1.25-5 μg/mL for 2 h in IPEC-J2 cells), whereas the present study employed 50 μM for 48 h. Differences in exposure duration and concentration may substantially influence quercetin’s redox behaviour and downstream cytokine responses, potentially explaining the divergent IL-8 outcome observed here. Although quercetin is widely recognized for its anti-inflammatory properties, higher concentrations or prolonged exposure have been associated with pro-oxidant activity, which may alter redox-sensitive signalling pathways and cytokine regulation [71,72]. In the present study, the concurrent elevation of ROS and IL-8 at 50 μM quercetin suggests a possible association, potentially involving ROS-sensitive signalling pathways such as NF-κB; however, this mechanistic link was not directly examined. Importantly, vehicle controls did not induce IL-8 production, and lower quercetin concentrations had no stimulatory effect, making solvent effects or generalized endotoxin contamination unlikely explanations for the concentration-specific IL-8 increase observed. Finally, the observation that quercetin at 25 μM enhanced IL-8 production in the presence of high-dose PHA suggests that quercetin may interfere with the regulatory networks that suppress chemokine responses during strong T-cell stimulation.

These results highlights that the immunomodulatory effects of quercetin are complex and context-dependent rather than uniformly inhibitory. While our research was focused on cytokine production, the precise molecular intermediaries such as p38 MAPK, NF-κB and Nrf2 pathways were not directly quantified. Future research utilizing Western blotting or pathway-specific inhibitors is required to confirm whether these classical pathways are the primary drivers of quercetin’s effects in porcine immune cells. Moreover, as our experiments only focused on two inflammatory markers (IL-6 and IL-8), a broader cytokine panel, including TNF-α, IL-1β, and IL-10, would be necessary to fully characterize the immunomodulatory profile of quercetin, particularly at higher concentrations.

Finally, it is important to highlight that while our study provides valuable preliminary insights into the effects of quercetin on porcine PBMCs, using only 3 pigs as donor animals poses a limitation, as this small sample size may not fully capture the biological diversity present in larger pig populations. Although the observed reduction in ROS and changes in IL-6 and IL-8 reached statistical significance, these findings should be interpreted as exploratory, and further studies with a larger cohort are warranted to validate these results and to explore the impact of age, breed and sex on the porcine immune response to quercetin. A further limitation of our study is the smaller number of technical replicates in some experiments (e.g., 48 h cytokine measurements). Therefore, additional studies including a larger number of animals and technical replicates would be valuable to further evaluate inter-individual variability in cytokine responses.

## 5. Conclusions

Our results demonstrate that porcine PBMCs respond to diverse stimuli (LPS, ConA, and PHA) with increased production of ROS, IL-6, and IL-8, supporting their utility as a physiologically relevant large-animal immune model for studying oxidative stress and inflammation. While these findings highlight their value in species-specific investigations and suggest potential translational relevance, direct comparison with human PBMC responses will be necessary to further substantiate cross-species applicability. In our experiments, quercetin behaved as an antioxidant while not displaying clear anti-inflammatory effects. Further studies evaluating quercetin’s effect on other cytokines (e.g., IL-1β, IL-10, TNF-α) are needed to map out the immunomodulatory effects of this flavonoid on pPBMCs. Due to their methodological characteristics, only limited conclusions can be drawn from the results of our in vitro experiments. Nevertheless, our data provide a valuable starting point for planning further research, including in vitro and in vivo experiments aimed at investigating the mechanism of action of quercetin and other flavonoids.

## Figures and Tables

**Table 1:** Treatment groups used for the measurement of cell viability.

**Control**	supplemented RPMI
**ST-LPS**	LPS derived from *Salmonella enterica* ser. Typhimurium L6143, 1 μg/mL
**SE-LPS**	LPS derived from *Salmonella enterica* ser. Enteritidis L770, 1 μg/mL
**EC-LPS**	LPS derived from *Escherichia coli* O111:B4, 1 μg/mL
**ConA-2.5**	concanavalin A, 2.5 μg/mL
**ConA-5**	concanavalin A, 5 μg/mL
**PHA-2**	phytohemagglutinin, 2 *V*/*V*%
**PHA-10**	phytohemagglutinin, 10 *V*/*V*%
**Q-25**	quercetin, 25 μM
**Q-50**	quercetin, 50 μM

**Table 2:** Combination treatment groups used for the evaluation of IC ROS, IL-6 and IL-8.

**ST-LPS + Q-25**	*S*. Typhimurium LPS, 1 μg/mL + quercetin, 25 μM	**ConA-2.5 + Q-50**	concanavalin A, 2.5 μg/mL + quercetin, 50 μM
**ST-LPS + Q-50**	*S*. Typhimurium LPS, 1 μg/mL + quercetin, 50 μM	**ConA-5 + Q-25**	concanavalin A, 5 μg/mL + quercetin, 25 μM
**SE-LPS + Q-25**	*S*. Enteritidis LPS, 1 μg/mL + quercetin, 25 μM	**ConA-5 + Q-50**	concanavalin A, 5 μg/mL + quercetin, 50 μM
**SE-LPS + Q-50**	*S*. Enteritidis, 1 μg/mL + quercetin, 50 μM	**PHA-2 + Q-25**	phytohemagglutinin, 2 *V*/*V*% + quercetin, 25 μM
**EC-LPS + Q-25**	*E. coli* LPS, 1 μg/mL + quercetin, 25 μM	**PHA-2 + Q-50**	phytohemagglutinin, 2 *V*/*V*% + quercetin, 50 μM
**EC-LPS + Q-50**	*E. coli* LPS, 1 μg/mL + quercetin, 50 μM	**PHA-10 + Q-25**	phytohemagglutinin, 10 V/V% + quercetin, 25 μM
**ConA-2.5 + Q-25**	concanavalin A, 2.5 μg/mL + quercetin, 25 μM	**PHA-10 + Q-50**	phytohemagglutinin, 10 V/V% + quercetin, 50 μM

## Data Availability

The original contributions presented in this study are included in the article. Further inquiries can be directed to the corresponding author.
